# Simultaneous imaging of magnetic field and temperature distributions by magneto optical indicator microscopy

**DOI:** 10.1038/srep43804

**Published:** 2017-03-02

**Authors:** Hanju Lee, Sunghoon Jeon, Barry Friedman, Kiejin Lee

**Affiliations:** 1 Department of Physics and Basic Science Institute for Cell Damage Control, Sogang University, Seoul 121-742, Republic of Korea; 2 Department of Physics, Sam Houston State University, Huntsville, TX 77341, USA

## Abstract

We report a simultaneous imaging method of the temperature and the magnetic field distributions based on the magneto optical indicator microscopy. The present method utilizes an optical indicator composed of a bismuth-substituted yttrium iron garnet thin film, and visualizes the magnetic field and temperature distributions through the magneto-optical effect and the temperature dependent optical absorption of the garnet thin film. By using a printed circuit board that carries an electric current as a device under test, we showed that the present method can visualize the magnetic field and temperature distribution simultaneously with a comparable temperature sensitivity (0.2 K) to that of existing conventional thermal imagers. The present technique provides a practical way to get a high resolution magnetic and thermal image at the same time, which is valuable in investigating how thermal variation results in a change of the operation state of a micrometer sized electronic device or material.

The magneto optical indicator microscopy (MOIM) is one of the most important techniques for the high resolution imaging of the magnetic field distributions and magnetic domain structure^1^. The MOIM detects a local magnetic field from the magneto-optical (MO) effects such as the magnetic circular dichroism (MCD) or the Faraday rotation (FR) effects of the MO indicator (MOI) placed on a device under test (DUT), and it visualizes the magnetic field distribution by using a conventional polarized light microscope system^1^,^2^. Because the MOIM is able to use a parallel optical sensor array such as the charge coupled device (CCD) camera, where each pixel of the CCD sensor detects a local MO signal at the same time, it has a unique advantage in the measurement throughput compared to other scanning magnetic field imagers such as the magnetic force microscope (MFM)^3^, superconducting quantum interference device microscope^4^ or the Hall-probe microscope^5^.

An important application of the MOIM is a visualization of an electric current distribution for the non-destructive testing and evaluation (NDT/E) for a micro-sized electronic device^6^. Because the desired object of the MOIM is typically an accurate sensing of the magnetic field distribution, most of the studies have made an effort to reduce the temperature change of a device under test during the measurement. However, the real operation condition of an electronic device always involves a change of temperature induced by electrical energy dissipation. In particular, characterization of a thermal property for a micro-scaled device is critical for evaluating the device performance, reliability, and durability^7^,^8^. Therefore, if the MOIM can visualize the temperature simultaneously with the magnetic field distribution, it could be an excellent measurement platform that provides rich information to test and evaluate a miniaturized modern electronic device.

Recently, a novel scheme for the high resolution thermal imaging based on the MOIM system using a pyro-magneto-optical (PMO) indicator was reported^9^. This method uses a MO material whose magnetic phase transition temperature is around room temperature, and detects a temperature variation from a change of the FR effect, where the change of the FR effect is caused by an alteration of saturation magnetization of the MO material near the magnetic phase transition. In the above reference, the authors claim that the PMO indicator approach will be straightforward for the simultaneous imaging of temperature and magnetic field. However, an experimental demonstration implementing the simultaneous imaging was not presented. Particularly, it is unclear how the thermal and the magnetic field image can be constructed separately from the MO signal when the temperature and the magnetic field changes at the same time. Because the thermal and magnetic field sensing are based on the same physical quantity, that is, the magnetization parallel to the light propagation direction, the change of the temperature and magnetic field can result in an identical variation of the MO signal. Therefore, utilizing individual physical effects for the temperature and magnetic field sensing would be a more straightforward way for the simultaneous imaging of the temperature and magnetic field.

Bismuth substituted yttrium iron garnet (Bi-YIG) is well known material for the MOI fabrication^10^,^11^. The Bi-YIG has an outstanding magneto optical property at the visible wavelength^10^,^11^, and it can be prepared as a thin film form on an inexpensive amorphous substrate such as glass^12^. Recently, it was reported that the yttrium iron garnet materials displayed a marked thermo-chromic effect that the color changes continuously from green to brownish, which offers potential application as a temperature indicator^13^. Therefore, one can expect that the magnetic field and temperature change can be visualized at the same time by analyzing the optical intensity change caused by the MO and the thermo-chromic effect. In present study, we show that the MOIM based on the Bi-YIG material can visualize the temperature and magnetic field distribution simultaneously from the MO effect and temperature dependent optical absorption caused by the thermo-chromic effect of the Bi-YIG.

## Results

### Measurement Principle

**Figure 1:**
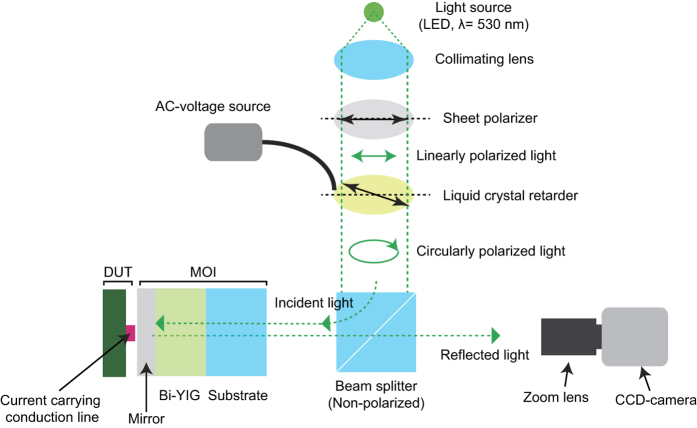
Illustration of the measurement setup. A MOI composed of a magneto optical (MO; bismuth substituted yttrium iron garnet) and a mirror (aluminum thin film; thickness:100 nm) layers coated on a glass substrate was placed on a device under test (DUT), and monitored by polarization microscope system with a light emitting diode (LED, λ = 530 nm) light source. The linearly polarized light by a sheet polarizer was modulated to be the left-handed and the right-handed circularly polarization states by a variable liquid crystal retarder. The optical axis of the liquid crystal retarder was aligned by 45° to the polarization direction of the incident light, and the retardation was modulated by changing the applied AC voltage. The modulated light propagated to the magneto optical indicator (MOI) placed on the DUT through a non-polarized beam splitter, and the light intensity reflected from the mirror layer was measured by a CCD camera.

Figure 1 illustrates the experimental setup and measurement principle. The MOI, which is composed of a MO (Bi-YIG thin film) and a mirror layer, is placed on an electric circuit and is monitored by a polarization microscope system based on a typical CCD camera. The linearly polarized incident light is modulated into the left-handed and right-handed circularly polarization (LHCP, RHCP) states by the variable liquid crystal retarder, and the modulated probe light propagates to the MOI placed on a DUT through the beam splitter. When an electric current flows along the conduction line, a local magnetic field generated by the current changes the magnetization state of the Bi-YIG thin film. For circularly polarized states of a probing light, the optical absorption is a function of a magnetization oriented parallel to the direction of light propagation. This is known as the magnetic circular dichroism (MCD)^14^, which is the differential absorption of left and right circularly polarized (LCP and RCP) light. At the same time, the local temperature is changed by the Joule heating by the electric current, and as a result, the optical absorption is changed by the thermo-chromic effect of the Bi-YIG material. In this condition, the intensity of the light passing through the MOI depends on the polarization state of the light, magnetization direction of the Bi-YIG thin film, and temperature dependent optical absorption of the Bi-YIG thin film. Then, one can express the light intensity as^15^:


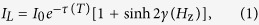



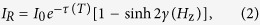



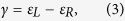


where *I*_0_ is the incident light intensity, *I*_L_ and *I*_R_ are measured light intensity for the left- and right-handed circularly polarized states, τ is the optical absorption coefficient of the MOI, *T* is the temperature, *H*_z_ is the magnetic field parallel to the light propagation direction, and *ε*_L_ and *ε*_R_ are the extinction coefficients of the Bi-YIG for the LCP and RCP light, respectively. For a linear regime (Δ*τ* ≪ 1, Δ*γ* ≪ 1), one can calculate the magnetic field and temperature from the change of optical intensity by the following equations:


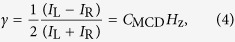



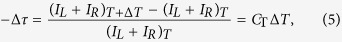


where, *H*_z_ is the magnetic field parallel to the propagation direction of the light, *C*_MCD_ and *C*_T_ are the MCD coefficient and temperature dependent absorption coefficient of the Bi-YIG material.

### Simultaneous Imaging Results

**Figure 2:**
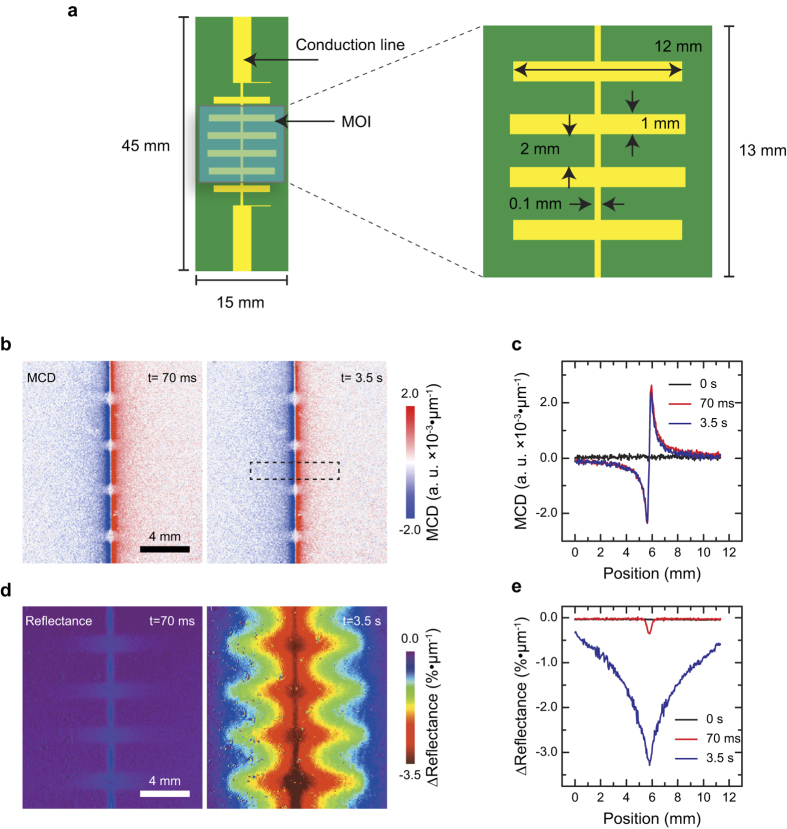
Experimental results for the simultaneous imaging of magnetic field and temperature distribution. (**a**) Illustrations of the printed circuit board (PCB) with the magneto optical indicator (MOI) used as the device under test (DUT). The yellow parts indicate patterned conductor (material: copper; thickness: 20 μm), green parts indicate the substrate (material: FR4; thickness = 1 mm) of the PCB, and a blue rectangle indicates the magneto optical indicator (MOI). (**b**) Magnetic circular dichroism (MCD) images at 70 ms and 3.5 s after applying an electric current of 7 A to the DUT. The MCD images were calculated by using the eq. (4) from optical images measured by the CCD camera. (**c**) Line profiles of the MCD images around a region showing an intense MCD signal (indicated by a dashed rectangle in **b**). (**d**) Calculated absorption images using by eq. (5) at 70 ms and 3.5 s. (**e**) Line profiles of the calculated absorption images.

To verify the present idea, we conducted experiments for the simultaneous imaging of magnetic and temperature distribution imaging for a PCB circuit. Figure 2a illustrates the PCB circuit used in the experiment, 2b and 2d show MCD and optical absorption images measured at 70 ms and 3.5 s after applying a DC current to a PCB circuit, and 2c and 2e show line profiles of the images. The MCD images showed negative and positive peaks at the left and right sides of the narrow conducting wires, and the peaks appeared soon after the electric current was applied with a negligible change over time. The MCD peaks only appeared around the narrow conducting wires; this is because that the local magnetic field perpendicular to the MOI surface is strong around those regions. On the other hand, the optical absorption images showed an overall decrease of reflected optical intensity around the metallic parts of the PCB circuit, and showed a strong dependence on the elapsed time. This result indicates that the optical absorption of the Bi-YIG material is strongly dependent on the temperature, because the magnetic field generated by the DC current is constant as observed in the MCD images.

For the Bi-YIG material, the concentration of the bismuth strongly influences the magneto optical properties and the optical absorption of the material^16^,^17^,^18^. In particular, the optical properties strongly depend on the wavelength of the incident light, and therefore, it is critical to find a proper concentration of the bismuth and a wavelength of the probing light maximizing the measurement sensitivity. To verify the magnetic field and temperature sensitivity depending on the bismuth concentration and wavelength of probing light, we conducted a series of experiments by using MOIs with bismuth concentration (*x*) of *x* = 1.0 and *x* = 2.0 for three representative wavelengths, red (*λ* = 625 nm), green (*λ* = 530 nm), and blue (*λ* = 470 nm).

**Figure 3:**
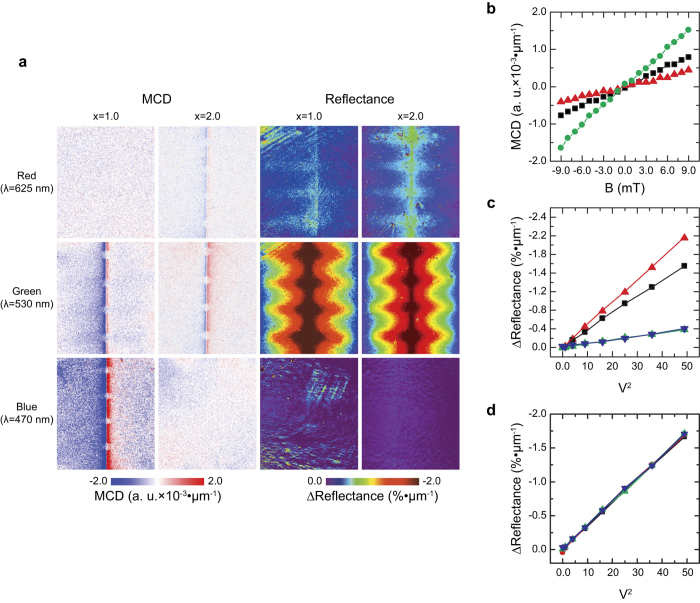
Experimental results for the simultaneous imaging of magnetic field and temperature distribution for various wavelengths of probing light and MOIs. (**a**) MCD and absorption images for three selected wavelengths of probing light (red: *λ* = 625 nm; green: *λ* = 530 nm; blue: *λ* = 470 nm) for MOIs with bismuth concentrations of 1.0 and 2.0. (**b**) Changes of MCD signals as a function of applied magnetic field perpendicular to the MOI surface: x = 2 MOI at *λ* = 530 nm (black line); x = 1 MOI at *λ* = 530 nm (red line); x = 1 MOI at *λ* = 470 nm (green line). (**c**) Optical absorption changes as a function of applied electrical power to the PCB circuit: x = 1 MOI at *λ* = 530 nm (red line); x = 2 MOI at *λ* = 530 nm (black line); x = 2 MOI at *λ* = 625 nm (green line); gold thin film at *λ* = 530 (blue line). (**d**) Optical absorption changes as a function of applied electrical power under external static magnetic field: 0 mT (black line); 22 mT (red line); 45 mT (green line); 67 mT (blue line).

Figure 3a shows measurement results on the MCD and absorption. From the measurement results, one can see that the green-wavelength light (*λ* = 530 nm) is suitable for the simultaneous probing the magnetic field and temperature, and the MOI of *x* = 1 provides a stronger MCD and absorption signals compared to that of *x* = 2. Although the MCD signal is maximized at the blue wavelength light (*λ* = 470 nm) for *x* = 1 MOI, there was no observable change in the absorption. Therefore, the blue wavelength light with *x* = 1 MOI will not be suitable for the simultaneous temperature and magnetic field distribution imaging. In addition, we note that the absence of the MCD signal of x = 2 MOI at *λ* = 470 nm comes from a strong absorption of the blue wavelength light. It is known that for the visible wavelength light, the MCD of Bi-YIG material is maximized around *λ* = 500 nm, and it is increased along with an increase of the bismuth concentration^16^,^17^,^18^. Although the increase of bismuth concentration enhances the MCD of the Bi-YIG material, it involves a red shift of the absorption edge that results in a rapid increase of the optical absorption at a short wavelength^16^,^17^,^18^. Because the MCD signal depends on the reflected light that has passed through the Bi-YIG thin film, the rapid increase of optical absorption will result in a decline and disappearance of the MCD signal.

### Magnetic Field Sensitivity

To quantify the measurement sensitivity on the magnetic field, we conducted MCD measurements as a function of magnetic field strength. Figure 3b shows the measurement results for *x* = 1 MOI with *λ* = 470 nm and *x* = 1, 2 MOIs with *λ* = 530 nm. The MCD signals increased linearly as the magnetic field strength increased in all case, but they had a different slope. The sensitivity can be calculated from the noise equivalent signal (NES) as ref. 19:


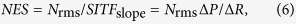


where *SITF*_slope_ is a slope of the system intensity transfer function (SITF) that describes the change of system response (Δ*R*) with respect to the change of an experimental parameter (Δ*P*), *N*_rms_ is the standard deviation of the fluctuation of the measured signal that comes from the CCD camera and an intensity fluctuation of the probing light. In present case, the *N*_rms_ and Δ*R* correspond to the fluctuation and change of the optical intensity measured by the CCD-camera, and Δ*P* corresponds to the external magnetic field.

The calculated *N*_rms_ and *SITF*_slope_ for 1000-averaged image were presented in the Table 1, where we calculated the *SITF*_slope_ of the x = 1 and x = 2 MOIs for a given unit thickness (1 μm) of the Bi-YIG layer. This is because that the strengths of the MO and the absorption signals depend on the thickness of the Bi-YIG layer. We note that the thickness dependence of the response signal strength (Δ*R*) implies that the uniformity of the thickness of the Bi-YIG layer is critical for the spatial temperature and magnetic field imaging. For present MOIs, the Bi-YIG layer was prepared by the metal organic decomposition (MOD) method with spin coating. It was known that the MOD method with spin coating deposition is capable to prepare a uniform Bi-YIG thin film^20^,^21^, and thus, we assumed that the prepared thin films had a uniform thickness (further detailed discussion on the thickness uniformity is presented in the Supplementary Information S1).

The calculated magnetic field sensitivity of MOIs: 0.031 and 0.028 mT•μm for the x = 1 at *λ* = 470 nm and *λ* = 530 nm; 0.063 mT•μm for the x = 2 MOI at *λ* = 530 nm, where the thickness dimension in the sensitivity unit indicates the thickness dependency of the MOI sensitivity. The calculated sensitivities were comparable to the magnetic field sensitivity of the MOIM reported previously^2^,^22^. This result indicates that *λ* = 530 nm with x = 1 MOI is most sensitive to the magnetic field imaging, although the change of the MCD signal of the MOI was maximized at *λ* = 470 nm. This is because of an enhancement of the noise (*N*_rms_) at *λ* = 470 nm, and it can be explained due to the increase of absorption at *λ* = 470 nm resulting in a decline of reflected light passed through the Bi-YIG layer. This result is consistent with the previous reports that the Bi-YIG material has a high figure of merit at the green-wavelength light for the magnetic field sensing due to its high transparency at that wavelength^16^.

### Temperature Sensitivity

The temperature sensitivity was calculated from the results for a PCB circuit measured as a function of applied electrical power. In addition, we conducted the same experiments by using a gold thin film (thickness: 100 nm) coated on a glass substrate as the indicator to compare the temperature sensitivity. The gold thin film is useful to estimate the temperature sensitivity of the MOIs because it has a high thermoreflectance coefficient at the green wavelength light^23^. Figure 3c shows the change of a local reflectance of MOIs and the gold thin film as a function of applied electrical power to the PCB circuit. The reflectance of the MOIs and gold thin film decreased linearly along with an increase of applied power with different slopes depending on the bismuth concentration and wavelength of the probing light. From the temperature measured by a thermocouple, the calculated thermo-reflectance coefficient of the gold thin film was −3.63 × 10^−4^ K^−1^, which was slightly larger than the known value (−2.36 × 10^−4^ K^−1^)^23^. This overestimation could be the result of the temperature being measured by the thermocouple being slightly smaller than the real temperature of the PCB circuit. We calculated temperature sensitivity by calculating the noise equivalent temperature difference (NETD)^19^ from equation (6) and from the slopes of the reflectance change (shown in Table 2), where the calibrated temperature from the thermo-reflectance measurement of the gold thin film was used for the calculation. For 1000-averaged averaged images, the calculated NETD of the gold thin film and MOIs were: 1.12 K for gold thin film at *λ* = 530 nm; 0.18 K•μm and 0.27 K•μm for x = 1 and x = 2 MOIs at *λ* = 530 nm; 1.06 K•μm for x = 2 MOI at *λ* = 625 nm. Therefore, we can conclude that the temperature sensitivity of the MOI with x = 1 at *λ* = 530 nm is around 0.2 K•μm, and this value is comparable to that of modern thermal imagers (35–200 mK)^7^,^8^,^24^,^25^,^26^,^27^.

To explain the physical origin of the increase of the optical absorption by an increase of temperature, more study will be required investigating the detailed spectroscopic properties and electronic structure of the Bi-YIG material, and it is beyond the scope of the present study. As shown in Fig. 3a, the change of absorption of the Bi-YIG has a strong dependency on the wavelength of the light. In particular, the change of the optical absorption was not influenced by an external magnetic field strength applied on the MOI as shown in Fig. 3d, and the MCD was not influenced by the temperature change as shown in Fig 2b,c. These results imply that the change of optical absorption by a temperature change is not related to the magneto optical property of the Bi-YIG material, and therefore, the simultaneous imaging on the temperature and magnetic field can be achieved.

## Discussion

In conclusion, we report the optically resolved simultaneous imaging method on the magnetic field and temperature distributions by Bi-YIG based MOIM system. Our experimental results showed that the optimized measurement performance was achieved by using the MOI of 1.0 bismuth concentration and the probing light of *λ* = 530 nm. For 1000-times averaged measurements, the measurement sensitivity for the magnetic and temperature was 0.3 mT•μm and 0.2 K•μm, respectively, and these were comparable to that of conventional MOIM and thermal imagers. The present technique could provide a practical way to characterize how the thermal variation results in a change of the operation state of a micrometer sized electronic device and material, which is a critical issue in electronics and material science.

## Materials and Methods

### Fabrication of magneto optical indicator

Bismuth yttrium iron garnet thin films were prepared on a glass substrate by the metal organic decomposition method (MOD)^2^,^10^,^20^. The MOD solutions (purchased from Kojundo Chemical Laboratory) with compositions of *x* = 1 (Bi:Y:Fe = 1:2:5; concentration: 4%) and *x* = 2 (Bi:Y:Fe = 2:1:5; concentration: 4%) were deposited by the spin-coating method with 3000 rpm for 30 s, and after then, the films were dried and annealed at 100 °C and 400 °C, respectively. The coating, drying, and annealing processes were repeated 20 times, and finally, the coated films were crystallized by a high temperature annealing process: 750 °C for 1-hour for the *x* = 1 film; 640 °C for 3-hours for the *x* = 2 film. The prepared Bi-YIG thin films were coated by the aluminum thin film (thickness: 100 nm) through the vacuum thermal evaporation technique. The Bi-YIG film thickness was measured by cross-section analysis through the field emission scanning electron microscopy (FESEM) as shown in Supplementary Figure S1a,b. The measured thickness of the Bi-YIG thin films were around 800 nm. Further details on the preparation method are presented in the references^2^,^10^,^12^,^20^.

### Imaging system

The imaging system consisted of a charge coupled device (CCD) based polarized light microscope, where light emitting diodes (LDE) with a wavelength of 470 nm, 530 nm, and 625 nm were used as the light source, and a liquid crystal modulator (LCM) was used to modulate the polarization state of the incident light to be circularly polarized states^28^. The incident light was polarized linearly by a sheet polarizer, and after then, the linearly polarized light was modulated into the left-handed and right-handed circularly polarized light by changing the AC voltage applied to the LCM. The reflected light from the MOI was monitored by a CCD camera, and the MCD and absorption images were calculated by the eq. (2) from the measured images by the CCD camera. All instruments were controlled through the serial and GPIB communications by a personal computer with a custom program.

## Additional Information

**How to cite this article:** Lee, H. *et al*. Simultaneous imaging of magnetic field and temperature distributions by magneto optical indicator microscopy. *Sci. Rep.* **7**, 43804; doi: 10.1038/srep43804 (2017).

**Publisher's note:** Springer Nature remains neutral with regard to jurisdictional claims in published maps and institutional affiliations.

## Supplementary Material

Supplementary Material

## Figures and Tables

**Table 1:** Parameters for the magnetic field sensitivity calculation.

Sample^a^	Wavelength (nm)	*N*_rms_^b^	*SITF*_slope_^c^ (mT^−1^•μm^−1^)	Sensitivity (mT•μm)
x = 1.0	530	2.5 × 10^−4^	2.03 × 10^−3^	0.028
x = 1.0	470	5.3 × 10^−4^	1.71 × 10^−2^	0.031
x = 2.0	530	2.9 × 10^−4^	4.58 × 10^−3^	0.063

^a^*x* is the concentration of bismuth of the Bi-YIG layer of the MOI.

^b^*N*_rms_ is the standard deviation of the averaged optical intensity measured by CCD camera.

^c^*SITF*_slope_ is the slope of the system intensity transfer function.

**Table 2:** Parameters for the temperature sensitivity calculation.

Sample^a^	Wavelength (nm)	*N*_rms_^b^	*SITF*_slope_^c^ (K^−1^•μm^−1^)	Sensitivity (K•μm)
x = 1.0	530	2.57 × 10^−4^	−1.46 × 10^−3^	0.18
x = 2.0	530	2.94 × 10^−4^	−1.09 × 10^−3^	0.27
x = 2.0	625	2.55 × 10^−4^	−2.41 × 10^−4^	1.06

^a^*x* is the concentration of bismuth of the Bi-YIG layer of the MOI.

^b^*N*_rms_ is the standard deviation of the averaged optical intensity measured by CCD camera.

^c^*SITF*_slope_ is the slope of the system intensity transfer function.
