# Supplementary material for: Simultaneous imaging of magnetic field and temperature distributions by magneto optical indicator microscopy
Source: Sci Rep. 2017 Mar 2;7:43804. doi: 10.1038/srep43804 (PMC5333159; doi:10.1038/srep43804)
Supplement: Supplementary Material [file srep43804-s1.pdf]

Simultaneous imaging of magnetic field and temperature distributions by magneto optical indicator microscopy

Hanju Lee[1], Sunghoon Jeon[1], Barry Friedman[2], and Kiejin Lee[1,*]

[1]Department of Physics and Basic Science Institute for Cell Damage Control, Sogang University, Seoul 121-742, Republic of Korea

[2]Department of Physics, Sam Houston State University, Huntsville, TX 77341, USA

*Corresponding Author: E-mail adress: klee@sogang.ac.kr (K. Lee)

SUPPLEMENTARY INFORMATION

S1. Measurement results and discussions on the uniformity of the thickness of the Bi-YIG thin film.

Supplementary figure S1a-b show the cross sectional image of the MOIs (x=1.0, 2.0) measured by the scanning electron microscope (SEM). The measurement results showed three distinct layers: the glass substrate (bottom), the Bi-YIG layer (middle), and the aluminum thin film layer (indicated by white arrow). The thickness of the Bi-YIG layer for x=1 and x=2 MOI were around 800nm. The variations of the film thickness were negligible in the measured area (~6 μm) for both MOIs.

Supplementary figures S1c-d show absorption image measured by using x=2.0 MOI and its line profiles along the vertical direction (indicated by dotted color lines in S1c). The line profiles showed a similar strength of absorption signals when they were positioned with the same distance (red and yellow: 2.2 mm; magenta and green: 3.6 mm) from the center signal line (cyan). In addition, supplementary figures S1e-f show MCD image and its line profiles along the horizontal direction (indicated by dotted color boxes in S1e). From the MCD line profiles (S1f), one can see that the strengths of the MCD signals are close to each

other. This result indicates that the gradient of the absorption line profile on the center signal line (cyan in S1c-d) comes from a real temperature gradient of the device under test rather than a gradient of the film thickness. From these results, we concluded that the prepared Bi-YIG layers had a uniform thickness.

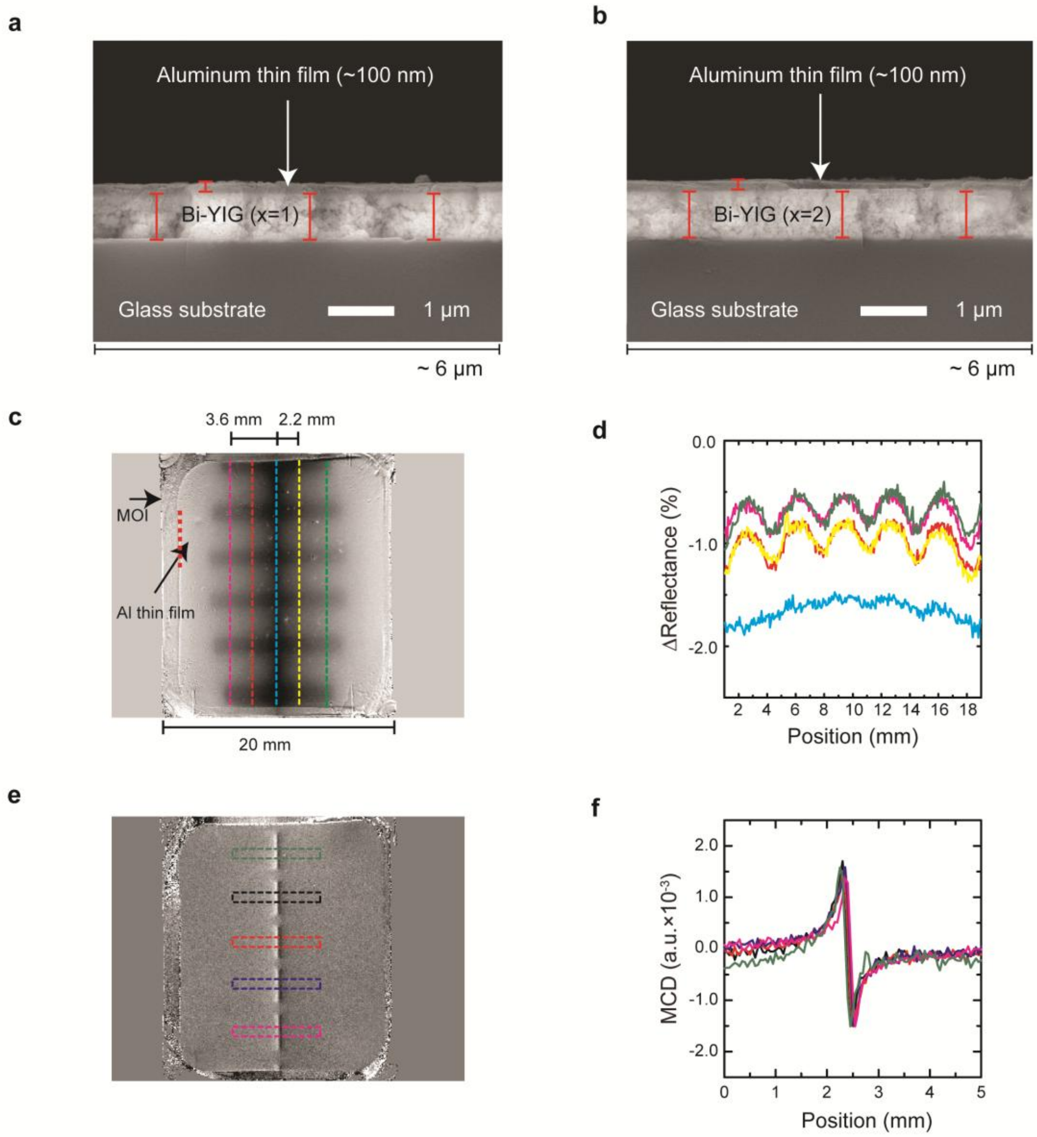

Supplementary figure S1: a-b. Cross section images of the MOIs (**a**:x=1, **b**:x=2) measured by the scanning electron microscope (SEM). **c-d** The absorption image and its line profiles along the vertical direction. The regions of the line profiles are indicated by dotted lines. **e-f** The MCD image and its line profiles along the horizontal direction. The regions of the line profiles are indicated by dotted line boxes.
